# Role of indocyanine green fluorescence and inferior thyroid artery ligation in hypocalcemia after total-thyroidectomy

**DOI:** 10.17305/bb.2025.12442

**Published:** 2025-06-25

**Authors:** Enrico Battistella, Luca Pomba, Silvia Rocchi, Giovanna Magni, Riccardo Toniato, Antonio Toniato

**Affiliations:** 1 Endocrine Surgery Unit, Department of Surgery, Veneto Institute of Oncology, IOV-IRCCS, Padua, Italy; 2 Clinical Research Unit, Veneto Institute of Oncology, IOV-IRCCS, Via Gattamelata 64, 35128 Padua, Italy; 3 School of Medicine, University of Padua, Via Giustiniani 2, 35128 Padua, Italy

**Keywords:** Total thyroidectomy, hypocalcemia, transient hypoparathyroidism, permanent hypoparathyroidism, indocyanine green fluorescence, inferior thyroid artery

## Abstract

Postoperative hypocalcemia remains a frequent complication of total thyroidectomy, and identifying intraoperative strategies to prevent it continues to be a key clinical challenge. This retrospective study aims to evaluate the role of indocyanine green (ICG) fluorescence imaging and truncal ligation of the inferior thyroid artery in the development of hypocalcemia following total thyroidectomy. Additionally, it seeks to identify factors associated with or predictive of hypocalcemia measured on postoperative day 1, as well as to determine the prevalence of transient and permanent hypoparathyroidism. The study included 200 patients who underwent total thyroidectomy performed by the same surgical team between January 2023 and March 2024. The surgical technique employed involved ligation of the inferior thyroid artery at its proximal trunk. Serum calcium levels were assessed for all patients during hospitalization, and again at 10 and 30 days post-surgery. Additionally, the relationship between postoperative calcemia and the vascularization of at least one parathyroid gland was evaluated using ICG fluorescence imaging. Hypocalcemia (<8.5 mg/dL) was observed in 81 patients (40.5%) on postoperative day 1. The rate of transient hypoparathyroidism was 31% (62 patients), while permanent postoperative hypoparathyroidism was seen in 2.5% (5 patients). Patients with a positive vascularization index on ICG fluorescence imaging were significantly less likely to experience hypocalcemia (Fisher’s exact test, *P* ═ 0.0014). The truncal ligation of the inferior thyroid artery did not significantly influence the occurrence of transient or permanent hypoparathyroidism. In conclusion, ICG fluorescence imaging is confirmed as a useful tool for evaluating parathyroid vascularity, although its associated costs should be carefully considered.

## Introduction

Approximately 40,000 thyroidectomies are performed each year in Italy with a two-night hospitalization and high efficiency thanks to the low rate of complications, the low level of post-operative pain and the rapid recovery of patients [1, 2]. Despite the undoubted socio-economic benefits of early discharge, the greatest concerns are the difficulty of predicting and treating possible complications. The three major post-thyroidectomy complications are compressive hematoma, single-lateral recurrent paralysis dysphonia or bilateral recurrent paralysis asphyxia crisis, and, finally, post-operative hypocalcemia.

Hypocalcemia seems to be the least dramatic complication but is the most insidious, as it can appear in mild-asymptomatic or severe-symptomatic forms. It is defined by calcium values <8.5 mg/dL related to albumin concentration and can be caused by vascular damage or due to the accidental or unavoidable removal of several glands, as the parathyroids have large functional reserves [3, 4].

Total thyroidectomy has the greatest impact on the development of this complication because it can cause vascular damage, burns or exeresis of the parathyroids. Post-surgical hypoparathyroidism can be transient or permanent. The need for calcium and active vitamin D analogues up to six months after thyroid surgery is defined as transient post-surgical hypoparathyroidism; while the need after six months is defined as permanent hypoparathyroidism. The rates of occurrence reported for this complication are 40%–60% for the transient from (at least 60% of which is resolved within 4–6 weeks), and 4%–25% for permanent hypoparathyroidism [3–8].

Transient hypoparathyroidism includes acute post-surgical hypoparathyroidism, which refers to immediate post-operative hypocalcemia that develops in the first days after surgery. Although this complication seems completely harmless, it is an important element that contributes to delaying the hospital discharge of patients after surgery, and, especially in its severe forms, reduces the quality of life of patients, due to the possible symptomatology that can even be life threatening. Post-operative hypocalcemia can be detected in the days following surgery by a blood test and confirmed by any symptoms reported by the patient. However, hypocalcemia does not always meet a parathyroid hormone (PTH) value under the range, and some studies have also shown that a decrease in calcemia of 1 mg/dL compared with the pre-operative value is associated with hypocalcemia symptoms [7, 8].

The literature confirms indocyanine green fluorescence imaging as a useful tool to assess vascularization of the parathyroids gland after total thyroidectomy and to predict the possible development of acute post-operative hypocalcemia [9–11].

Indocyanine green (ICG) is the first substance discovered to emit fluorescence in the near-infrared (NIR) spectrum and its use in medicine has been approved by the Food and Drug Administration (FDA) in 1959 for the evaluation of liver and heart functions. Its use in the surgical field has become increasingly widespread today thanks to the introduction of specific optical systems. It is widely used in general surgery, from colorectal surgery to biliary surgery, and is increasingly taking hold in endocrine surgery, both for thyroid and parathyroid pathologies and for adrenal pathologies. ICG fluorescence imaging can be used to assess the vascularity of parathyroid glands at the end of thyroid surgery and therefore seems to be able to predict acute hypocalcemia after surgery [11].

This study includes patients who underwent total thyroidectomy in the Endocrine Surgery Unit at the Veneto Institute of Oncology. According to the standard protocol, during the hospital stay—which is usually two-three days long—the patients’ therapy was adjusted (oral or intravenous calcium/vitamin D), based on their blood chemistry results. Some patients were also administered ICG for intra-operative evaluation of parathyroid vascularity.

This study aims to verify the impact of proximal truncal ligation of the inferior thyroid artery and other factors that can be associated with or predict hypocalcemia measured on post-surgery day 1 and assess the prevalence of transient or permanent hypoparathyroidism. In addition, the vascularity of the parathyroids was evaluated in patients who were administered ICG and related to the appearance of post-operative hypocalcemia.

## Materials and methods

### Patients and study design

All adults who underwent total thyroidectomy at our center between January 2023 and March 2024 were included in this retrospective observational study. Patients with a medical history of prior thyroid and parathyroid surgery or a concurrent parathyroid disease (e.g., primary, secondary, or tertiary hyperparathyroidism) were excluded. The selected patients underwent total thyroidectomy (standard, retrosternal or with latero-cervical lymphadenectomy) with open technique and by the same endocrine surgery team. All these data are analyzed considering the surgical technique applied in our facility. The technique involves the proximal truncal ligation of the inferior thyroid artery.

When intra-operative administration of green of indocyanine was possible, the procedure included an intravenous injection of a bolus of ICG after the total thyroidectomy. The amount of ICG injected was precisely calculated based on the patient’s weight at a fixed dose of 0.2 mg/kg. After a few seconds, all the lights in the room were turned off and the surgeon subjectively evaluated parathyroid perfusion with a score ranging from 0 (vascularity absent) to 2 (good vascularity) using the available fluorescence system (IMAGE 1 S, KARL STORZ).

For each patient, the following data were included in the analysis: demographics (age and sex), body mass index (BMI), presence of thyroiditis, osteo-metabolic and cardiovascular co-morbidity, thyroid pathology (multi-nodular goiter, Graves’ disease, cytologically indeterminate nodules, thyroid carcinoma, toxic goiter), surgery of choice (total thyroidectomy, total retrosternal thyroidectomy, and total thyroidectomy with central lymphadenectomy), number of parathyroid glands seen with the naked eye, histological examination, thyroid volume removed, presence of parathyroid glands in the specimen.

**Figure 1.**
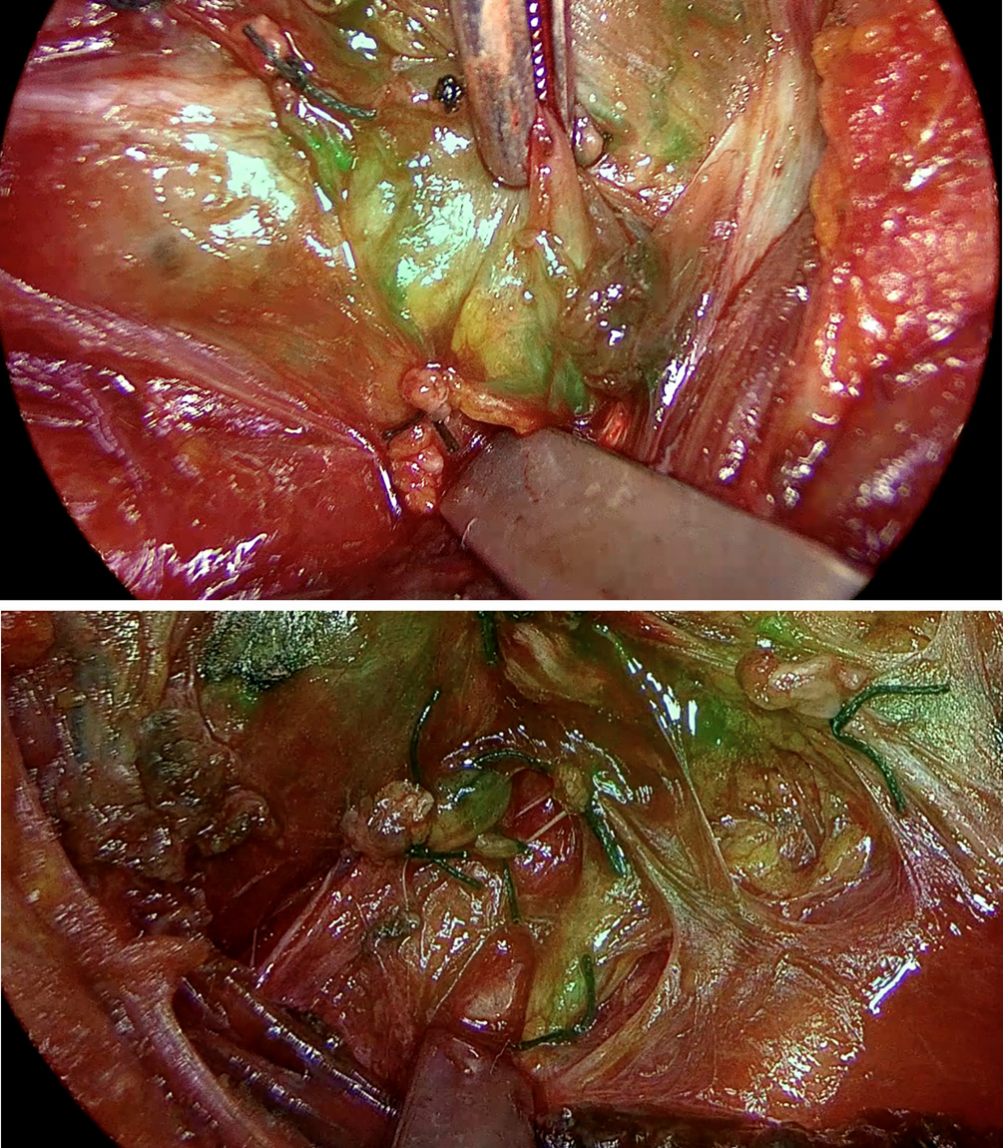
Example of indocyanine green fluorescence of the parathyroid gland at the end of a total thyroidectomy with ligation of the inferior thyroid artery.

In addition to the above data, we also included: use of ICG fluorescence imaging Yes/No (if Yes: number of parathyroid glands displayed, and vascularization score 0–2 by using ICG fluorescence imaging), the prevalence of hypocalcemia in the first post-operative day (<8.5 mg/dL), the prevalence of acute hypocalcemia in the first post-operative day (<8 mg/dL), the prevalence of transient hypoparathyroidism (the need for calcium and vitamin D therapy at the time of discharge) and the prevalence of permanent hypoparathyroidism (the need for calcium and vitamin D therapy 6 months after surgery). Furthermore, the use of the ICG fluorescence also identified viable parathyroid tissue with proximal ligation of the inferior thyroid artery (Figure 1).

According to the protocol, in the days following the surgery a calcium sample blood test was performed and appropriate therapy adjustments (calcium and vitamin D) were made based on the results of the test. In the study, calcemia was measured in mg/dL in the first, second, and, if still present, on the third day after surgery.

Depending on the calcemia values, different calcium dosages were then administered:

Calcemia < 8 mg/dL: 3 g of calcium carbonate + 1.5 mcg of calcitriol per day;

8 < calcemia <8.4 mg/dL: 1.5 g calcium carbonate + 1.5 mcg of calcitriol per day;

8.5 < calcemia < 8.9 mg/dL: 1 g calcium carbonate + 1 mcg of calcitriol per day.

The therapy was modulated following the above schedule until the day of discharge. Patients were examined at 7 days and 30 days of the surgery by means of a blood test. The therapy was maintained until the normalization of the calcemia. All the patients reassessed serum calcium levels at 6 months remaining in the follow-up.

### Ethical statement

This study was conducted in accordance with Good Clinical Practice (GCP) guidelines and the Declaration of Helsinki on human experimentation. It was also conducted in accordance with local regulations and guidelines. This retrospective observational study was approved by the Ethics Committee of the Veneto Oncology Institute IOV-IRCCS on July 29, 2024 (IOV-ENDCH-01-2024).

### Statistical analysis

All the variables collected were described: qualitative variables, orderable or classed by frequency distribution (absolute and percentage), and quantitative variables described by position indices (mean, median, quartiles, and standard deviation).

Data were described by dividing the cases according to the presence of hypocalcemia, transient hypoparathyroidism, and permanent hypoparathyroidism.

Chi-Square tests and Fisher’s tests were used to check for differences in frequency distributions for qualitative variables or classes. Student’s *t*-tests or non-parametric Wilcoxon or Krusckal Wallis tests were used for quantitative variables. The calculated tests were considered statistically significant when *P* < 0.05. Statistical Analysis System (SAS) software was used, version 9.4 (SAS Institute Inc., SAS Campus Drive, Cary, NC, USA).

In order to achieve the main objective, the number of subjects who showed hypocalcemia (blood calcium <8.5 mg/dL) on the first day after surgery was considered as a proportion of the total number of subjects who underwent total thyroidectomy. This percentage was accompanied by a 95% confidence interval (CI). The same procedure was applied to assess transient hypoparathyroidism in terms of the need for calcium and vitamin D therapy on the day of discharge and to assess permanent hypoparathyroidism in terms of the need for calcium and vitamin D six months after surgery.

## Results

From January 2023 to March 2024, 380 patients underwent thyroid and parathyroid surgery. Among them, 200 underwent total thyroidectomy. The mean age of surgical patients was 55.7 years (range: 18.1–85.5 years), with 156 females (78%) and 44 males (22%).

Patients were stratified by BMI: 6 underweight patients (3%) with BMI <18.4 kg/m^2^; 95 normal weight patients (47.5%) with BMI between 18.5 and 24.9 kg/m^2^; 63 overweight patients (31.5%) with BMI between 25 and 29.9 kg/m^2^; and 36 obese patients (18%) with BMI ≥30 kg/m^2^.

When comparing patients with and without ICG) fluorescence imaging, no significant differences were found: the female-to-male ratio was 3:1 in both groups; median age was 53.7 years vs 56.2 years; and median BMI was 24.1 kg/m^2^ vs 26.5 kg/m^2^, respectively.

Remote pathological history variables included: thyroiditis (positive autoantibodies) in 92 patients (46%); cardiovascular comorbidities requiring treatment in 82 patients (41%); prior vitamin D supplementation for deficiency in 59 patients (29.5%); and a history of breast cancer in 12 patients (6%).

**Table 1:** Data and statistical analyses of patients with hypocalcemia (<8.5 mg/dL) on post-operative day 1, with transient hypoparathyroidism and permanent hypoparathyroidism

	**Patients with hypocalcemia in POD1 (*n* = 81, 40.5%)**	**Patients with hypocalcemia on the day of discharge - transient hypoparathyroidism (*n* ═ 62, 31%)**	**Patients with hypocalcemia 6 months after surgery - definitive hypoparathyroidism (*n* ═ 5, 2.5%)**	***P* value**
Median age (years)	53.4	54.9	38.9	^*^0.2250
Sex (*n*)	Female ═ 71 (4.5%) Male ═ 10 (22.7%)	Female ═ 52 (83.9%) Male ═ 10 (16.1%)	Female ═ 5 (100%) Male ═ 0 (0%)	^§^0.0086
Thyroiditis (*n*)	39 (42.4%)	30 (48.4%)	2 (40%)	^§^0.6656
BMI (kg/m^2^)	25.8	25.2	24.6	^#^0.3189
Cardiovacular disease (*n*)	48 (59.2%)	35 (56.4%)	/	^§^0.8986
Indication for surgery				^#^0.5139
Multinodular goiter (*n*)	31 (38.3%)	27 (43.5%)	1 (20%)	
Basedow disease (*n*)	15 (18.5%)	13 (20.9%)	/	
Cytologically indeterminate nodule (*n*)	20 (24.7%)	11 (17.7%)	1 (20%)	
Thyroid carcinoma (*n*)	15 (18.5%)	11 (17.7%)	3 (60%)	
Type of surgery				^#^0.6288
Total thyroidectomy (*n*)	35 (43.2%)	32 (51.6%)	2 (40%)	
Retrosternal totale thyroidectomy (*n*)	22 (27.2%)	20 (32.3%)	/	
Total thyroidectomy with central lymphadenectomy (*n*)	24 (29.6%)	10 (16.1%)	3 (60%)	
Hystological report				^§^0.4594
Benign (*n*)	53 (65.4%)	41 (66.1%)	1 (20%)	
Malignant (*n*)	28 (34.6%)	21 (33.9%)	4 (80%)	
Thyroid volume (cc)	51.8 +− 60,4 (min ═ 5, max ═ 427)	32.3 (min ═ 5.5, max ═ 520)	26.6 (min ═ 22.6, max ═ 34.5)	^*^0.9059
Symptomatic patients (*n*)	13 (6.5%)	8 (12.9%)	3 (60%)	<0.000

^*^Kruskal–Wallis Test, ^#^Chi squared test, ^§^Fisher’s test. POD1: Post-operative day 1.

Surgical indications were: multinodular goiter in 74 patients (37%), Graves’ disease in 30 (15%), single indeterminate nodules in 53 (26.5%), and thyroid carcinoma in 43 (21.5%). In cases of benign disease, total thyroidectomy was performed due to compressive symptoms, dysphagia, or single indeterminate nodules with thyroiditis.

Thyroiditis was present in 52% of patients in the ICG group and 53% in the non-ICG group. No differences were observed in surgical indications; frequencies were evenly distributed between groups.

The surgical procedures performed included total thyroidectomy in 95 patients (42.5%), total retrosternal thyroidectomy in 55 (27.5%), and total thyroidectomy with central lymphadenectomy in 50 (27%).

Focusing on the primary aim of the study, hypocalcemia (serum calcium <8.5 mg/dL) occurred in 81 patients (40.5%) on postoperative day 1. Severe hypocalcemia (<8.0 mg/dL) was observed in 33 patients (16.5%).

Thirteen patients (6.5%; 95% CI: 3.5%–9.9%) experienced hypocalcemia symptoms such as paresthesia in the limbs or perioral region on postoperative day 1. Four patients (2%) required intravenous calcium gluconate, and in three (1.5%), symptoms persisted for three days postoperatively.

Transient hypoparathyroidism—defined as the need for calcium and vitamin D therapy at discharge—was observed in 62 patients (31%). Permanent hypoparathyroidism—defined as the need for calcium and vitamin D therapy six months after surgery—was present in 5 patients (2.5%).

Statistical analysis confirmed significant associations between both paresthesia and hypocalcemia on postoperative day 1 and the development of permanent hypoparathyroidism (*P* < 0.0001 for both). Extended hospital stays were also associated with increased risk of permanent hypoparathyroidism.

Length of hospital stay was as follows: two days in 121 cases (60.5%), three days in 73 (36.5%), and four days in 6 (3%).

Histological examination assessed histotype, number of parathyroid glands removed, and thyroid gland volume. Histological diagnoses included: 66 papillary carcinomas (33%), 7 follicular carcinomas (3.5%), 2 medullary carcinomas (1%), 3 NIFTP (1.5%), 24 follicular adenomas (12%), 97 nodular hyperplasia (48.5%), and 1 Hurthle cell carcinoma (0.5%).

Parathyroid glands were unintentionally or necessarily removed in 19 cases (one gland in 16 cases; two in 3 cases). The average thyroid gland volume was 51.2 cc (range: 5–525 cc).

Intraoperative identification of parathyroids by direct vision showed: two glands in 9 cases (4.5%), three glands in 50 cases (25%), and four glands in 141 cases (70.5%). ICG imaging was used to evaluate vascularization in 79 patients (39.5%). Among these, two glands were identified in 5 cases (6.3%), three in 40 (50.6%), and four in 34 (43.1%). No difference was observed between parathyroid gland counts by direct vision and ICG imaging.

Statistical analysis indicated hypocalcemia was significantly more frequent in female patients (Fisher’s test ═ 0.0086). No significant correlation was found between hypocalcemia and age (Kruskal–Wallis: *P* ═ 0.2250), BMI (Fisher’s test: *P* ═ 0.8747), or preoperative vitamin D intake (Chi-square: *P* ═ 0.3189).

The type of surgery (total thyroidectomy, retrosternal, or with lymphadenectomy) was not predictive of hypocalcemia (Chi-square: *P* ═ 0.6288). Similarly, no correlation was found between hypocalcemia and histotype or gland volume (Kruskal–Wallis: *P* ═ 0.9059) (Table 1).

A significant inverse relationship was observed between the number of parathyroid glands identified by direct vision and the incidence of hypocalcemia (Mantel-Haenszel Chi-square: *P* ═ 0.0251), confirmed by ICG fluorescence imaging (Table 2).

**Table 2:** Statistical analyses of the number of parathyroids identified with naked eyes and the presence of hypocalcemia in POD1, transient hypoparathyroidism and definitive hypoparathyroidism

**Number of parathyroids identified macroscopically**	**Patients with hypocalcemia in POD1 (*n* ═ 81, 40.5%)**	**Patients with hypocalcemia on the day of discharge-transient hypoparathyroidism (*n* ═ 62, 31%)**	**Patients with hypocalcemia 6 months after surgery - definitive hypoparathyroidism (*n* ═ 5, 2.5%)**
2	5 (6.2%)	2 (3.2%)	2 (40%)
3	21 (25.9%)	25 (40.3%)	2 (40%)
4	55(67.9%)	35 (56.5%)	1 (20%)
*P* value	0.0103	0.016485	/

Parathyroid vascularity evaluation showed that patients with at least one parathyroid scoring 2 (indicating preserved vascularization) were less likely to develop hypocalcemia (Chi-square: *P* ═ 0.0014). The sensitivity and specificity of ICG fluorescence in detecting viable parathyroids were 66.7% (CI: 50.77%–79.50%) and 73.1% (CI: 64.15%–80.46%), respectively (Table 3).

**Table 3:** ICG score and the presence of hypocalcemia in POD1, transient hypoparathyrodism and definitive hypoparathyroidism

	**Patients with hypocalcemia in POD1 (*n* ═ 32, 40.5%)**	**Patients with hypocalcemia on the day of discharge - transient hypoparathyroidism (*n* ═ 12, 15.2%)**	**Patients with hypocalcemia 6 months after surgery - definitive hypoparathyroidism (*n* ═ 2, 2.5%)**
No parathyroid with ICG score 2	18 (56.2%)	10 (83.3%)	2 (100%)
At least 1 parathyroid with ICG score ═ 2	14 (43.8%)	2 (16.7%)	0
P value (Fisher test)	0.0014	0.0028	>0.5

## Discussion

Postoperative hypocalcemia is the most frequent complication following total thyroidectomy [1, 2]. It is typically transient, resulting from gland manipulation, and only occasionally progresses to permanent hypoparathyroidism.

In this study, the day-1 postoperative hypocalcemia rate was 40.5% (<8.5 mg/dL), with severe cases (<8.0 mg/dL) at 16.5%. Symptomatic hypocalcemia occurred in 6.5%, with symptoms persisting in 1.5% of cases for more than three days.

Most patients undergoing total thyroidectomy are expected to be discharged on postoperative day 2, provided they show no hypocalcemia symptoms. Transient hypoparathyroidism was observed in 31% (62 patients). At the 10-day follow-up, therapy was discontinued in 25 patients, and after 30 days, calcium levels normalized in all except 5.

These findings are consistent with the literature. Definitions of post-surgical hypoparathyroidism vary widely, affecting reported prevalence. Guidelines from the European Society of Endocrinology report rates of 30%–60%, while a meta-analysis by Sanabria et al. cites 6%–55% [4, 7, 8, 12]. Differences are attributed to varying definitions, cutoff values, and the day post-surgery on which hypoparathyroidism is assessed [8–12].

Permanent hypoparathyroidism was observed in 5 patients (2.5%), which is lower than the range reported in current guidelines and consistent with the lower end of findings from similar studies [3, 4]. All five cases involved female patients. Although normocalcemic at 30 days post-op without treatment, symptoms later returned. In three cases, patients had received radioiodine therapy two months post-surgery; one had osteoporosis after chemotherapy/radiation for breast cancer; and another had accidental excision of two parathyroids during central neck dissection.

These results demonstrate that our surgical technique—ligating the inferior thyroid artery at the trunk rather than distally at the peri-capsular site, as suggested in multiple studies—does not increase the risk of either transient or permanent hypoparathyroidism [3, 13, 14]. This approach offers two key advantages in thyroid surgery: it reduces the risk of post-operative bleeding, thereby minimizing the chance of major hemorrhage. In 2023 at our Center, the rate of post-operative bleeding requiring emergency reoperation was just 0.32% (1 out of 312 thyroid procedures), significantly lower than rates reported in the literature [2, 15].

A second advantage of ligating the artery at the trunk is the improved clarity of the surgical field, which facilitates the safer identification of the recurrent laryngeal nerve and better preservation of the parathyroid glands.

In our study, we assessed parathyroid gland vascularization after thyroidectomy using ICG fluorescence imaging. Results showed that 53 patients (66.3%) had at least one well-vascularized gland (ICG score 2), and 12 patients (15%) had two well-vascularized glands. Additionally, all patients had at least two parathyroid glands with partial vascularization (ICG score 1). If parathyroid vascularization were supplied solely by the inferior thyroid artery—assisted only by the superior thyroid artery due to their anatomical connection—no fluorescence would be expected.

This finding aligns with prior studies, such as that by Johansson et al., which used laser Doppler fluorimetry to demonstrate that ligation of the inferior thyroid artery does not significantly impair parathyroid vascularization. This confirms that only part of the parathyroid blood supply depends on the inferior thyroid artery [16–18].

We emphasize the importance of preserving parathyroid viability by avoiding excessive dissection and retaining surrounding soft tissue—including adipose and connective tissue—containing small vessels from the pharyngeal, laryngeal, and tracheal planes, as recommended by several authors [19–20].

Various factors have been reported in the literature as contributing to hypocalcemia, transient hypoparathyroidism, and permanent hypoparathyroidism. Yamashita et al. identified female sex, young age, large thyroid goiter, prolonged surgery, excessive intraoperative blood loss, and low preoperative plasma calcium levels as key risk factors [20]. Proye et al. cited diffuse toxic goiter, advanced malignancy, total thyroidectomy with lymphadenectomy, and retention of fewer than three parathyroid glands [5, 6, 21]. Tartaglia et al. highlighted the importance of early post-operative thyroid hormone replacement therapy to prevent hypocalcemia. Maintaining a euthyroid state is essential, as thyroid hormones activate vitamin D, which is necessary for intestinal calcium absorption—even when parathyroid hormone (PTH) levels are normal [22].

In our study, multiple variables were analyzed. Female sex was the only factor significantly associated with hypocalcemia on postoperative day 1 (Fisher’s test: *P* ═ 0.0086). However, it was not significantly associated with transient or permanent hypoparathyroidism. The number of identified and preserved parathyroid glands was also statistically significant (Mantel-Haenszel Chi-square: *P* ═ 0.0251); as the number of preserved glands increased, the likelihood of developing hypocalcemia on day 1 decreased. This is consistent with findings by Sciumè et al., who emphasized that preserving the glands is critical, and that surgeon experience is a key factor in avoiding complications [23, 24].

Despite systematic efforts, parathyroid identification is not always successful. In our experience, incidental or necessary removal does occur. Histological analysis showed that in 16 cases (8%), one parathyroid was removed; in three cases (1.5%), two glands were excised. Among these, two patients with a single removed gland developed severe acute hypocalcemia on day 1 - one requiring IV calcium. One patient who had two glands excised also developed severe hypocalcemia.

Among patients who received ICG, 52 had at least one well-vascularized parathyroid (ICG score ═ 2). In this group, 14 patients (26.9%) developed hypocalcemia on postoperative day 1. Statistical analysis revealed that patients with at least one well-vascularized gland were significantly less likely to develop hypocalcemia (Fisher’s test: *P* ═ 0.0014). The test showed a sensitivity of 66.7% (95% CI: 50.77%–79.50%) and a specificity of 73.1% (95% CI: 64.15%–79.50%).

Despite the clear benefits of ICG fluorescence imaging in predicting post-operative hypocalcemia, cost remains a concern: each vial of ICG costs around €160, and the fluorescence imaging device approximately €10,000. The learning curve for using ICG is short, typically around 10 procedures, after which surgical time increases by no more than five minutes. This depends on team familiarity and scrub nurse turnover. As suggested in the literature, we advocate for ICG use particularly in centers where the imaging system is also applied to other surgical fields (e.g., hepatobiliary, gastric, and colorectal surgery) to justify and distribute the cost [10, 25]. Once validated, this technique could reduce hospital stays by enabling safe early discharge and decreasing readmission rates. However, as of now, clear economic benefits remain unproven.

## Conclusion

This retrospective observational study investigated risk factors for hypocalcemia following total thyroidectomy. The presence of symptomatic hypocalcemia may be a predictor of transient or permanent hypoparathyroidism. Our findings show that female sex and the number of visible parathyroids are predictors of hypocalcemia, but most notably, ligating the inferior thyroid artery at the trunk does not increase the risk of post-operative hypoparathyroidism.

ICG imaging is confirmed as a valuable tool to assess parathyroid vascularization, though cost and implementation considerations must be taken into account. These findings are particularly relevant for high-volume endocrine surgery centers aiming to reduce complication rates.

Prospective, multicenter studies are needed to validate these results and explore new therapeutic strategies. Future research should also incorporate additional confounding variables and leverage ICG imaging, using objective tools to better evaluate parathyroid viability and vascularization.

## Data Availability

Data found in the patients’ medical records.
